# Dedicated Industrial Oilseed Crops as Metabolic Engineering Platforms for Sustainable Industrial Feedstock Production

**DOI:** 10.1038/srep22181

**Published:** 2016-02-26

**Authors:** Li-Hua Zhu, Frans Krens, Mark A. Smith, Xueyuan Li, Weicong Qi, Eibertus N. van Loo, Tim Iven, Ivo Feussner, Tara J. Nazarenus, Dongxin Huai, David C. Taylor, Xue-Rong Zhou, Allan G. Green, Jay Shockey, K. Thomas Klasson, Robert T. Mullen, Bangquan Huang, John M. Dyer, Edgar B. Cahoon

**Affiliations:** 1 Department of Plant Breeding, Swedish University of Agricultural Sciences, Box 101, SE 230 53, Alnarp, Sweden; 2 Wageningen UR Plant Breeding, P.O. Box 386, 6700 AJ Wageningen, The Netherlands; 3 National Research Council of Canada, 110 Gymnasium Place, Saskatoon, SK S7N 0W9, Canada; 4 Department of Plant Biochemistry, Albrecht-von-Haller-Institute for Plant Sciences, Georg-August-University, Justus-von-Liebig-Weg 11, 37077, Göttingen, Germany; 5 Department of Plant Biochemistry, Goettingen Center for Molecular Biosciences (GZMB), Georg-August-University, Justus-von-Liebig-Weg 11, 37077, Göttingen, Germany; 6 Department of Plant Biochemistry, International Center for Advanced Studies of Energy Conversion (ICASEC), Georg-August-University, Justus-von-Liebig-Weg 11, 37077, Göttingen, Germany; 7 Center for Plant Science Innovation and Department of Biochemistry, University of Nebraska-Lincoln, Lincoln, NE 68588, USA; 8 National Key Lab of Crop Genetic Improvement and College of Plant Science and Technology, Huazhong Agricultural University, Wuhan 430070, China; 9 CSIRO Food & Nutrition, North Ryde, Sydney, NSW, Australia; 10 CSIRO Agriculture, Canberra, ACT, Australia; 11 U.S. Department of Agriculture, Agricultural Research Service, Southern Regional Research Center, Commodity Utilization Research Unit, New Orleans, LA 70124, USA; 12 Department of Molecular and Cellular Biology, University of Guelph, Guelph, Ontario N1G 2W1, Canada; 13 College of Life Science, Hubei University, Wuhan 430062, P. R. China; 14 U.S. Department of Agriculture-Agricultural Research Service, U.S. Arid-Land Agricultural Research Center, Maricopa, AZ 85138, USA

## Abstract

Feedstocks for industrial applications ranging from polymers to lubricants are largely derived from petroleum, a non-renewable resource. Vegetable oils with fatty acid structures and storage forms tailored for specific industrial uses offer renewable and potentially sustainable sources of petrochemical-type functionalities. A wide array of industrial vegetable oils can be generated through biotechnology, but will likely require non-commodity oilseed platforms dedicated to specialty oil production for commercial acceptance. Here we show the feasibility of three Brassicaceae oilseeds crambe, camelina, and carinata, none of which are widely cultivated for food use, as hosts for complex metabolic engineering of wax esters for lubricant applications. Lines producing wax esters >20% of total seed oil were generated for each crop and further improved for high temperature oxidative stability by down-regulation of fatty acid polyunsaturation. Field cultivation of optimized wax ester-producing crambe demonstrated commercial utility of these engineered crops and a path for sustainable production of other industrial oils in dedicated specialty oilseeds.

Development of a bio-based economy requires sustainable sources of hydrocarbon-type molecules to supplement or replace non-renewable petrochemicals for production of polymers, lubricants, and engine fuels. With declining petroleum sources and a rapidly expanding world population, the identification of renewable materials to support the bio-based economy will become increasingly imperative. Oilseed-derived vegetable oils and their constituent fatty acids are sources of renewable materials for industrial applications. Historically vegetative oils derived from oilseed crops such as soybean and rapeseed have been used primarily for human consumption as frying oils, margarines, salad oils, and a variety of other food products^1^,^2^. Given the high energy density of their fatty acid components, vegetable oils have been increasingly used for production of biofuels, including biodiesel and more recently jet fuel^2^,^3^,^4^. Vegetable oils also have many chemical and physical properties that make them suitable feedstocks for production of a variety of industrial materials currently derived from petroleum^2^,^3^,^5^.

The increasing availability of genetic resources coupled with advances in biotechnology and synthetic biology make the expanded use of vegetable oils as petroleum substitutes in non-edible applications a reality. A wide diversity of novel fatty acid structures and variant oil storage forms occur in plants and other organisms that can greatly expand the functional properties for industrial applications of crop-sourced vegetable oils^3^,^6^. Techniques are now well established for transfer of biosynthetic pathways for these novel fatty acids and oils from non-agronomic species to existing or novel oilseed crops to achieve commercial production. Perceived public and regulatory concerns and liability issues associated with unintended mixing of seeds for edible and industrial markets^7^,^8^, however, limit commercial interest in transgenically-produced industrial oils. Moreover, while strong identity preservation chains are in place to prevent this, any disruptions in these chains could have severe global impacts on agricultural commodity markets.

To preclude such problems, a need exists to establish dedicated non-food oilseed platforms for the metabolic engineering of novel industrial oils. In this report, we examine the use of three Brassicaceae oilseed species for production of wax esters, a class of industrially useful oils: *Crambe abyssinica* (crambe), *Brassica carinata* (carinata), and *Camelina sativa* (camelina). Unlike oilseeds such as soybean and rapeseed, none of these crops is currently grown on a commodity-scale for edible oil production, and although niche food markets do exist for carinata and camelina oil, crambe oil is entirely used as an industrial oilseed because its oil is enriched in erucic acid (22:1), which is perceived to be a cardiovascular risk for humans^9^,^10^,^11^. In addition, carinata and camelina are receiving considerable interest in North America for their ability to be productive on land with limited rainfall and soil fertility, such as that found in portions of the Great Plains and U.S. Pacific Northwest that currently have limited oilseed cultivation^2^,^12^,^13^. Robust genetic transformation methods have also been developed for each of these crops that enable complex metabolic engineering for generation of new, high value industrial oil traits^13^,^14^,^15^,^16^.

As a demonstration of the value of these crops for industrial oil production, we have explored the feasibility of generating wax ester-type oils rather than the more common triacylglycerol-based vegetable oils by transfer of the appropriate genes to these crops. Wax esters consist of a fatty acid esterified to a fatty alcohol. These molecules occur throughout nature in forms such as beeswax, avian preen gland oils, and sperm whale oil. Sperm whale oil has been highly desired over centuries for applications, including most recently high-temperature lubricants such as transmission fluids^17^,^18^. The availability of this oil for industrial markets has largely disappeared due to bans on hunting of sperm whales. A possible substitute for sperm whale oil is jojoba (*Simmondsia chinensis)* oil, which is composed of wax esters containing 38 to 44 carbon chain-lengths that have similar chemical and functional properties as sperm whale oil with wax esters containing 32 to 36 carbon chain-lengths^17^,^19^,^20^. However, jojoba plants are adapted to arid areas, such as the desert regions in the southwest United States, which limits their commercial production. As a result, jojoba oil is relatively more expensive and used largely in lotions and shampoos^21^,^22^. As an alternative approach to generating sperm whale oil functionality in a cost effective way for lubricants, interest has been directed to the introduction of jojoba wax ester-biosynthetic genes into existing oilseed crops. Genes for jojoba-type wax ester biosynthesis have been previously identified and their use for the production of wax ester-rich oils has been demonstrated in seeds of the model plant *Arabidopsis thaliana*^23^,^24^. These genes include those encoding a fatty acyl-CoA reductase (FAR) that converts fatty acyl-CoAs into fatty alcohols^24^ and wax synthase (WS), an acyltransferase-like enzyme that esterifies fatty acyl-CoAs to fatty alcohols to form wax esters^23^. Here we describe the use of the three potential industrial oilseeds as hosts for seed-specific expression of *FAR*, *WS* and associated genes to tailor the optimized production of wax esters.

## Results

### Production of Wax Esters in Dedicated Industrial Oilseed Crops

**Figure 1:**
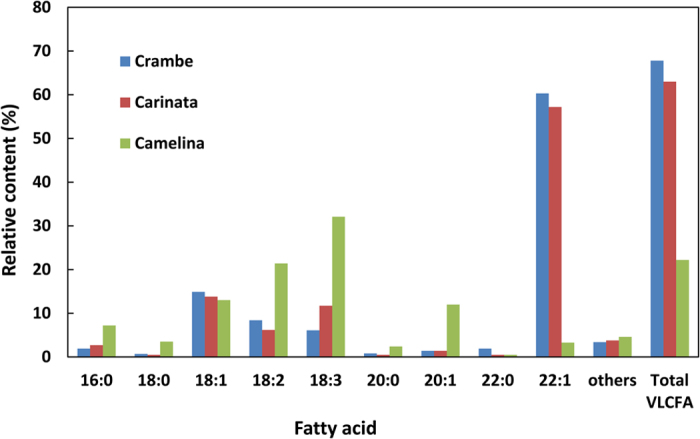
Comparison of the fatty acid compositions of seed oils from the genetic backgrounds of crambe (*Crambe abyssinica)*, carinata (*Brassica carinata)*, and camelina (*Camelina sativa)* that were used for metabolic engineering for wax ester production in this study. Data shown are for wild-type crambe and camelina seeds, while carinata data are for seeds engineered for *FAD2*-RNAi suppression and crambe *FAE1* overexpression to increase erucic acid from 38% of total fatty acids in wild type seeds to 58% of total fatty acids in seeds of lines used for metabolic engineering^25^.

Although crambe, carinata, and camelina are all members of the Brassicaceae family, their seed oils have distinct fatty acid compositions that impact metabolic engineering strategies for production of industrial oils. As shown in Fig. 1, crambe seed oil is comprised of almost 70% very long-chain fatty acids (VLCFA; ≥C20), including ~60% erucic acid (22:1). While wild-type carinata oil contains about 38% erucic acid (data not shown), in a previous study, genetic modification by silencing the carinata *FAD2* and co-expression of the *crambe FAE1* yielded stable transgenic lines containing up to 58% erucic acid line and almost 64% VLCFAs^25^. Camelina seed oil, by contrast, has only 20 to 25% VLCFA and <2.5% erucic acid and is instead enriched in α-linolenic acid (18:3), which comprises ~35% of the seed fatty acids (Fig. 1).

**Figure 2:**
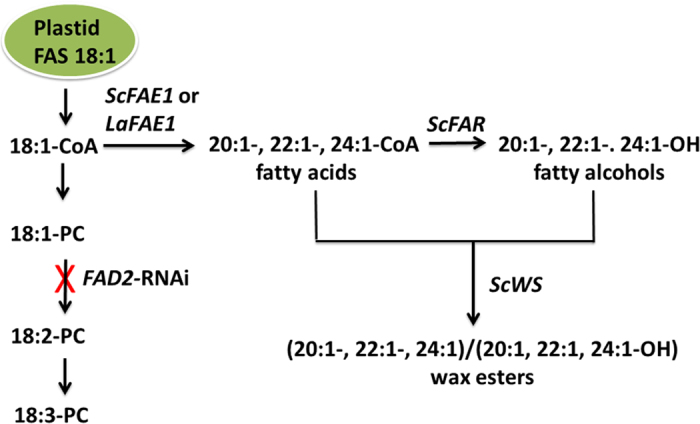
Metabolic engineering strategies for production of wax esters in the three target crops. Wax esters were produced by seed-specific expression of jojoba cDNAs for fatty acyl-CoA reductase (*ScFAR*) that converts fatty acyl-CoAs into fatty alcohols and wax synthase (*ScWS*), an acyltransferase-like enzyme that esterifies fatty acyl-CoAs to fatty alcohols to form wax esters. Oleic acid (18:1) synthesized from fatty acid synthase (FAS) localized in the plastid is the primary substrate for wax ester production. Pools of very long-chain fatty acids (VLCFAs; ≥C20) were increased by seed-specific expression of cDNAs for the enzymes 3-ketoacyl-CoA synthase-related fatty acid elongases from jojoba (*ScFAE1*) or *Lunaria annua* (*LaFAE1*). Levels of monounsaturated C18 substrate (18:1-CoA) for wax esters were increased by seed-specific RNA interference (RNAi) suppression of the *FAD2* gene (*FAD2*-RNAi) for the Δ12-oleic acid desaturase.

To demonstrate the feasibility of these three oilseeds as platforms for the design and biotechnological production of industrial oils, the wax ester biosynthetic pathway from jojoba was overlaid on to the existing triacylglycerol biosynthetic pathways of seeds of the three crops. Wax ester biosynthesis requires the esterification of a fatty acid from an acyl-CoA substrate to a fatty alcohol, effectively bypassing the typical incorporation of fatty acids onto glycerol backbones to form triacylglycerols (Fig. 2). To accomplish this reaction, jojoba *FAR* (*ScFAR*) and *WS* (*ScWS*) cDNAs were co-expressed under control of strong seed-specific promoters in the target crops. The jojoba wax ester biosynthetic pathway functions primarily with C20 and C22 fatty acyl-CoA substrates^23^,^24^, which is ideally suited for the fatty acid profile of crambe and carinata seeds. Because camelina seeds have relatively low VLCFA content (Fig. 1), a cDNA for the jojoba fatty acid elongase 1 (*ScFAE1*)-like 3-ketoacyl-CoA synthase was also introduced to increase VLCFA production as substrates for FAR and WS. For comparison, studies were also conducted in crambe and carinata with and without the inclusion of the jojoba *FAE1*-like enzyme. In addition, the host germplasm for carinata studies was a line optimized for production of wax esters with monounsaturated acyl chains, generated by suppression of the *FAD2* gene that controls the desaturation of oleic acid (18:1) to form linoleic acid (18:2) and overexpression of a crambe *FAE1*^25^. Transgene cassettes for *ScWS*, *ScFAR*, and *ScFAE1* were assembled in a single binary vector for transformation of the crops to increase the likelihood of simple, linked insertion patterns of the transgenes to simplify future breeding efforts and biotechnological introduction of additional transgenes.

**Figure 3:**
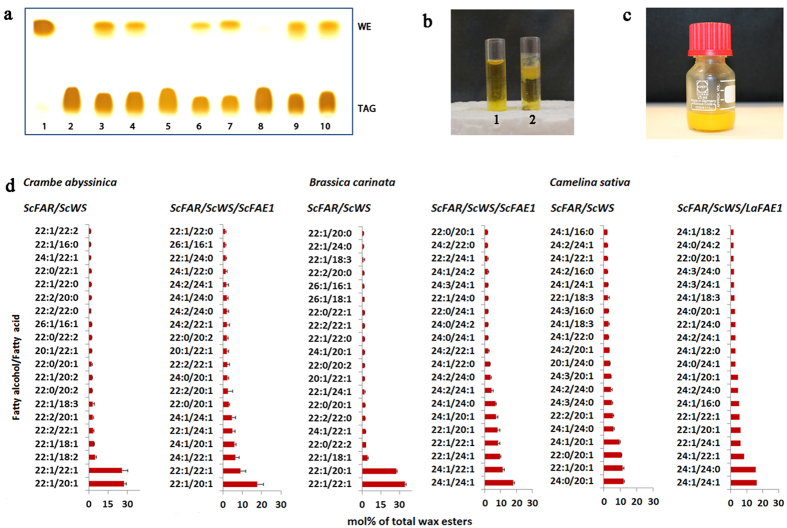
Wax ester (WE) production in the seeds of the three target crops. (**a**) Thin layer chromatographic (TLC) separation of triacylglycerol (TAG) and WE. [Lane 1 = standard WE from jojoba seed oil. Lanes 2, 5 and 8 show wildtype of the three respective crops and lanes 3, 4, 6, 7, 9 and 10 transformed crambe, carinata and camelina, respectively. 2–4 = *Crambe abyssinica*. 5–7 = *Brassica carinata*. 8–10 = *Camelina sativa*.] (**b**) Camelina oil from wild-type seeds (left, 1) and WE-producing seeds (right, 2) at 4 °C. The solid material in (2) is enriched in WE and demonstrates the higher melting point of WE versus TAG. (**c**) Crambe WE extracted from oil of engineered seeds. (**d**) Twenty most abundant WE molecular species in engineered seeds of the three oil crops. n = 3 to 5 single seed replicates (crambe, carinata) or replicates of 5 mg of seed (camelina) ± SD.

**Figure 4:**
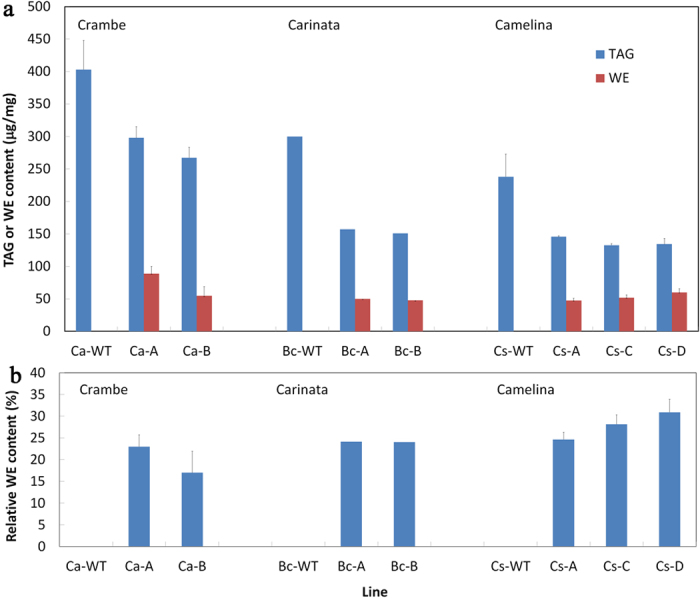
Absolute and relative content of triacylglycerol (TAG) and wax ester (WE) in in the seed oil of the three target species. Shown in (**a**) is the absolute content of TAG and WE expressed in μg/mg seed wt, and shown in (**b**) is the relative WE content expressed in percent of total TAG and WE content (n = 3 to 5 biological replicates ± SD). (**b**) Ca = *Crambe abyssinica*. Bc = *Brassica carinata*. Cs = *Camelina sativa*. WT = wild-type. A-D indicate lines engineered with different transgene combinations as follows: A, *ScFAR*/*ScWS*; B, *ScFAR*/*ScFAE1*/*ScWS*; C, *ScFAR*/*ScWS*/*LaFAE1*; D, *ScFAR*/*ScWS*/*LaFAE1/CsFAD2*-RNAi.

Analysis of lipid extracts by thin layer chromatography from seeds of ten to fifteen independent transgenic events for each crop indicated the successful production of wax esters (Fig. 3a–c). Total wax ester content of seeds measured by gas chromatography following hydrolysis of purified wax esters and triacylglycerol fractions revealed wax ester amounts of 15 to 30% of the total oil content (wax esters + triacylglycerols; Fig. 4a,b). Wax ester amounts of >50% of the total oil were detected in single seeds of the top performing crambe lines (Supplementary Table S1). Wax esters in seeds of the three crops had distinct fatty acid and fatty alcohol compositions. In both crambe and carinata seeds, wax esters were composed predominantly of 20:1 and 22:1 fatty acids and 22:1 fatty alcohols (Fig. 3d). This was reflected in the molecular species of wax esters produced in these seeds, which were enriched in C42 and C44 species consisting of 22:1 fatty alcohols esterified to 20:1 and 22:1 fatty acids. The wax esters of camelina seeds contained a more complex mixture of fatty acids and fatty alcohols, including high levels of 20:1, 22:0, 22:1, 24:0, and 24:1 fatty acids and 20:1, 22:0, 22:1, 24:0, 24:1, 24:2, and 24:3 alcohols (Fig. 3d). Of the fatty alcohols, C24 alcohols comprised nearly 50% of the total fatty alcohols of camelina wax esters. These combinations of fatty acids and fatty alcohols resulted in wax esters of primarily C46 and C48 chain-lengths. Similarly, the inclusion of the *ScFAE1* along with jojoba *ScFAR* and *ScWS* in crambe and carinata resulted in a shift to include higher content of C24 fatty acids and fatty alcohols (Fig. 3d), namely an increase in wax content of C46 and C48.

### Tailoring Wax Ester Structures By Step-Wise Metabolic Engineering of Fatty Acid Composition

**Figure 5:**
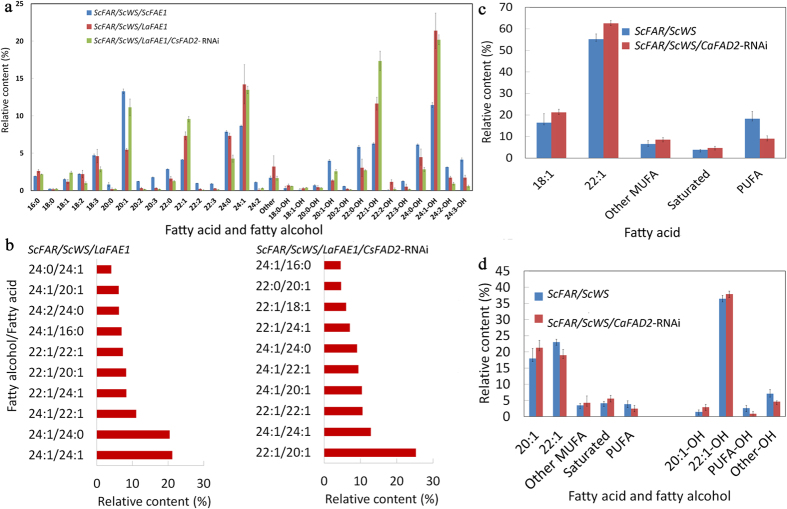
Tailored wax ester (WE) compositions by RNAi suppression of *FAD2* gene in camelina (**a,b**) and crambe seeds (**c,d**). Shown in (**a,b**) are data from seed wax esters of a camelina line engineered for expression of *ScFAR*/*ScWS*/*LaFAE1* +/− RNAi suppression of *FAD2* (*CsFAD2*-RNAi). Fatty acid and fatty alcohol components of wax esters (**a)** and ten most abundant wax ester species (**b**) in transgenic camelina lines. Shown in (**c,d**) are measurements of seed triacylglycerols (TAGs) and wax esters (WE) respectively of a crambe line engineered for expression of *ScFAR*/*ScWS* +/− genetic cross with a *FAD2* RNAi suppression line (*CaFAD2*-RNAi). TAG (**c**) and WE (**d**) compositions in crambe seeds before and after genetic crossing of a wax ester producing line with a *CaFAD2*-RNAi suppression line in comparison with the parental wax ester line. For data in (**a,c,d**) n = 3 to 5 biological replicates ± SD. MUFA, monounsaturated fatty acids; PUFA, polyunsaturated fatty acids.

Experiments described above in carinata and crambe indicated an influence of *ScFAE1* expression on wax ester composition. To explore this in more detail, step-wise metabolic engineering experiments were conducted in camelina, which is readily amenable to rapid gene testing due to its ability to be transformed with a simple *Agrobacterium*-based method using multiple selection markers and its short life cycle^16^. Initially, the *ScFAE1* cDNA was replaced in the construct with the *LaFAE1* gene from *Lunaria annua*. In contrast to the broad acyl-CoA substrate specificity of *ScFAE1*^26^, the *LaFAE1* displays higher activity *in vivo* for monounsaturated acyl-CoA substrates and is also effective in fatty acid elongation to the C24 chain-length^27^. Consistent with this, camelina seeds engineered for co-expression of the *LaFAE1* with the *ScFAR* and *ScWS* displayed a marked reduction in wax esters containing saturated and polyunsaturated fatty alcohols, relative to seeds expressing the *ScFAE1* (Fig. 3d). Most striking was a 2-fold increase in relative content of C22 and C24 monounsaturated fatty acids and alcohols in wax esters compared to those generated with the *ScFAE1* in the three-gene construct. This accumulation occurred in part at the expense of C20 monounsaturated fatty acids, which decreased by ~50% in seeds expressing the *LaFAE1*. Based on these findings, additional experiments were conducted to obtain further increases in wax esters containing monounsaturated fatty acids and alcohols for achieving wax esters with better oxidative stability at high temperatures. For these experiments, two of the camelina lines expressing *LaFAE1* and *ScFAR* and *ScWS* with the highest wax ester content were re-transformed with a seed-specific *FAD2-*RNAi transgene to reduce polyunsaturated fatty acid content and channel oleic acid (18:1) to wax ester fatty acids and fatty alcohols. This resulted in substantial increases in wax esters with monounsaturated 20:1 and 22:1 fatty acids and 22:1 fatty alcohols, as well as additional decreases in saturated and polyunsaturated wax ester components (Fig. 5a). These differences were also observed in the wax ester molecular species. The major wax esters in seeds expressing the *LaFAE1* were C48 species consisting of a 24:1 fatty alcohol paired with either a 24:0 or 24:1 fatty acid (Fig. 5b). With the additional suppression of *FAD2*, the major wax ester was instead a C42 species comprised of a 22:1 alcohol paired with a 20:1 fatty acid (Fig. 5b). The shift in relative content from 24:1 to 20:1 and 22:1 in wax ester fatty acids and fatty alcohols with *FAD2* suppression likely results from limiting fatty acid elongation flux for conversion of the increased pools of 18:1-CoA to 24:1-CoA.

This strategy was also applied in crambe by crossing the line expressing *ScFAR* and *ScWS* with the line expressing the *CaFAD2*-RNAi. The hybrid progenies showed an increased level of oleic acid (Fig. 5c), indicating the channeling of 18:1 to 18:2 was effectively reduced. Wax ester was detected in seed oils in the overwhelmingly majority of F_3_ seeds with proportions up to over 40 weight % of total oil (Supplementary Table S2). Similar to the results achieved in camelina reported above, a considerable reduction in seed oil polyunsaturated fatty acids and alcohols was observed in the seed oil, but 20:1 fatty acid, 20:1 alcohol and 22:1 alcohol increased (Fig. 5d). Overall, these results demonstrate that it is possible to alter fatty acid unsaturation for tailoring wax ester profiles by regulating the target gene expression in camelina and crambe.

### Agronomic Properties of Dedicated Industrial Oilseeds Engineered for Wax Ester Production

Transgenic wax ester crambe lines (T_5_ generation) were used for a confined field trial in Kristianstad, Sweden. Plants were grown under insect nets in the field to mitigate bee pollination as required by EC regulations. Transgenic lines with *ScWS* and *ScFAR* genes showed a slightly reduced seed yield, oil content and germination rate compared to the wild type (Table 1), while the transgenic line with the *ScWS*, *ScFAR* and *ScFAE1* genes had a dramatically reduced seed yield and oil content (Table 1a). In contrast, the thousand seed weight of both transgenic lines was higher than that of wild type (Table 1a). Except for delayed seedling establishment relative to the wild type, both transgenic lines had normal vegetative growth in the field. Flowering and fruit set were clearly delayed and reduced for the transgenic line containing three jojoba genes. Evaluation of agronomic traits of transgenic crambe (T_6_ generation) in greenhouses showed a similar tendency to that of the field trial, but the reduction in seed yield and oil content of the transgenic lines were much lower than that of the field trial (Table 1b). Normal seedling growth, seed-set and seed development were observed for the transgenic line with *ScFAR* and *ScWS* genes. Similar to the results from the field, the transgenic line with the *ScWS*, *ScFAR* and *ScFAE1* genes had weak seedling growth, poor seed-set, and delayed seed maturation relative to the wild type under the greenhouse conditions. Camelina lines expressing the *ScWS*, *ScFAR*, and *LaFAE1* +/− *FAD2* RNAi suppression also displayed reduced germination under greenhouse conditions, with germination rates of seeds from the transgenic lines ranging from 25 to 80% versus 94 to 100% in seeds from the wild type (Supplementary Fig. 1). Notably, germination rates were higher in wax ester-producing seeds with *FAD2* RNAi suppression versus wax ester-producing seeds in a wild type background.

## Discussion

Here we have demonstrated the feasibility of crambe, camelina, and carinata as biotechnological platforms for the seed-specific production of wax esters. Seed oils of crambe and carinata have the highest endogenous levels of very long-chain fatty acids of the three oilseeds evaluated, and yielded wax ester profiles that most closely mimic the C42-rich wax ester composition of jojoba seeds by seed-specific expression of only the jojoba *FAR* and *WS* genes. We have also shown that an array of different wax ester compositions can be generated by co-expression of *FAE1* genes from different sources and by channeling monounsaturated fatty acids to wax ester production by suppression of the *FAD2* ∆12 oleic acid desaturase gene (Fig. 2). Using these approaches, wax ester levels of >30% of the total oil content were achieved in seeds of the engineered crops.

Germination, seed yield per plant, and oil content of crambe seeds producing 22% to 25% wax esters from expression of jojoba *FAR* and *WS* showed no obvious effect in greenhouse and field studies (Table 1). However, seed germination was significantly compromised in crambe and camelina lines engineered for co-expression of an additional *FAE1* gene (Table 1, Supplementary Fig. S1). The common feature of these seeds was the accumulation of C48 wax esters species composed of C24 fatty acids and alcohols, which were nearly undetectable in crambe lines lacking the *ScFAE1* transgene. The striking difference in seed germination between lines lacking and containing the *ScFAE1* transgene may be due to physical properties of C48 wax esters, e.g. melting points that compromise seed performance. Alternatively, C48 wax esters or their C24 acyl components, such as fatty alcohols, may negatively affect metabolism (e.g. β-oxidation) during seed germination or maturation. Based on these studies, it appears that engineered seeds may be more tolerant towards the presence of shorter chain wax esters. This is consistent with data in Supplementary Fig. 1 showing that *FAD2* RNAi suppression in wax ester-producing camelina lines nearly doubled seed germination rates. Seeds from these lines have a relatively higher content of wax esters with 20:1 and 22:1 fatty acids and alcohols and lesser relative amounts of C24 fatty acids and alcohols compared to wax ester-producing seeds lacking *FAD2* RNAi suppression (Fig. 5A). In the case of camelina, a more effective metabolic engineering strategy may be to express a *FAE1* from a species such as Arabidopsis, *Teesdalia*, *Brassica napus* or *Crambe abyssinica* that produce high levels of 20:1 or 22:1 in their seeds along with *FAD2* suppression to generate shorter chain wax esters compared to that obtained from the *LaFAE1* or *ScFAE1* expression.

It is notable that jojoba seed oil is deficient in TAG, but instead consists almost entirely of wax esters^19^,^21^. By comparison, crambe lines selected for field testing have ~25% of the oil as wax esters, with the remainder in the form of TAG. It remains to be determined if this level of wax ester accumulation results in oil quality with industrial value. Since sperm whale wax esters were used as additives in petroleum based lubricants, it can be anticipated that the blend of TAG and wax esters produced in crambe will have superior lubrication properties compared to a pure TAG based lubricant. Alternatively, wax esters and TAG can be partitioned through methods such as winterization or crystallization based on the higher melting point of wax esters (as shown for winterized camelina oil +/− wax esters in Fig. 3b). In this way, value can be obtained from both the wax ester and TAG components of the engineered oil. Possible future metabolic engineering strategies to enrich for wax ester production include blocking triacylglycerol production via RNAi suppression of *DGAT1* and *PDAT1*, based on recent findings with Arabidopsis^29^.

Overall, the findings reported here demonstrate the feasibility of transferring multigenic biosynthetic pathways to dedicated non-food oilseed crops for biotechnological production of an industrial oil trait. In the case of wax ester production, crambe was particularly well-suited because of the high content of VLCFAs, mainly erucic acid, in its seeds. New transgenic crambe lines with increased erucic acid have since been developed^28^, which are likely to be a more suitable background for wax ester production. However, as shown with camelina and crambe, through the use of biotechnological techniques, the fatty acid compositions of seeds of these crops can be rationally tailored to provide pools of fatty acid substrates that are optimized for production of target industrial oil traits. For instance, as we have shown above, down-regulation of the *FAD2* gene in combination with overexpression of jojoba *FAR* and *WS* resulted in the production of wax esters in crambe seed oil consisting mainly of 20:1 and 22:1 monounsaturated fatty acids and fatty alcohols, which confers a higher oxidative stability amenable to formulations of motor oil and transmission fluids. Production of wax esters containing higher amounts of monounsaturated molecular species also allows for easier separation of wax and TAG components of seed oil, which reduces costs associated with downstream purification. Engineering of seeds for production of high value industrial proteins and/or small molecule co-products that can be readily partitioned from oils could offer additional revenue streams for establishing crambe, carinata, and camelina as economically viable dedicated industrial oilseed crops.

## Methods

### Plant materials

*Crambe abyssinica* cv. Galactica (crambe), *Camelica sativa* cv. Suneson (camelina), and *Brassica carinata* (carinata) were used in this study. For carinata, the XS18A 6–2 line engineered for high erucic acid and low polyunsaturated fatty acid seed oil content was used for transformation^25^. Transgenic crambe lines of all generations including non-transgenic control plants were grown in biotron or greenhouse. The conditions in the biotron included a photoperiod of 16 h at a light intensity of 250 μmol m^−2^ s^−1^, temperature of 21 ^o^C/18 ^o^C (day/night) and humidity of 60%. The greenhouse conditions were the 16h photoperiod at 21 ^o^C/13 ^o^C (day/night). Fertilizer (N:P:K = 21:3:10) was applied when plants were about 4 weeks old and thereafter once a week. All flowering plants were covered with plastic bags to prevent the potential hybridization between different lines.

Camelina plants were grown under greenhouse conditions with 14h day length (24 °C to 26 °C) and 10 h dark (18 °C to 20 °C) with natural and supplemental lighting at 400–500 μmol m^−2^s^−1^ as described previously^12^. All carinata transgenic and control lines were grown in the greenhouse under natural light conditions supplemented with high-pressure sodium lamps with a 16 h photoperiod (16 h of light and 8 h of darkness) at 22 °C and a relative humidity of 25 to 30%.

### RNA isolation from jojoba seeds and first strand cDNA synthesis

Jojoba (*Simmondsia chinensis*) developing and mature seeds were collected in an established jojoba plantation grown by New South Wales Department of Agriculture at Condobolin, New South Wales, Australia. The developing seeds were sorted visually into three classes, early maturity (small and juicy seeds), middle maturity (solid tissue) and late maturity (close to ripe seed). The embryos were dissected out from the seeds and stored in RNA *later* (Qiagen) solution for RNA extraction. Total RNA was extracted with TRIzol solution (Invitrogen) from the middle and late stage groups of developing embryos and precipitated with 0.5 vol isopropanol and 0.5 vol 3 M sodium acetate pH 5.2. The precipitate was washed with 75% ethanol. Air dried RNA was resuspended in 20 μl water. First strand cDNA was produced with the RevertAid H Minus Reverse Transcriptase (Fermentas/Thermo) according to manufacturer’s protocol.

### Preparation of transformation vectors

The following vectors were used in this study:1) pFWS2-Kan (*ScFAR* and *ScWS*); 2) pFWS3-Kan (*ScFAR*, *ScWS* and *ScFAE1*); 3) pFWS3 (*ScFAR*, *ScWS,* and *ScFAE1*); 4) pFWS3-LaFAE1(*ScFAR, ScWS* and *LaFAE1*); 5) pBinGlyBar1-CsFAD2-HP (*CsFAD2*-RNAi); 6) pFWS2-Hyg (*ScFAR* and *ScWS*) and 7) pFWS3-Hyg (*ScFAR, ScWS* and *ScFAE1*). Vectors 1 and 2 were used for crambe transformation with kanamycin as selection; vectors 2, 4–5 were used for camelina transformation with DsRed as a marker for screening; and vectors 6 and 7 were used for carinata transformation with hygromycin as selection.

#### Preparation of pFWS2-Kan, pFWS3, and pFWS3-Kan

First strand cDNA obtained above was used as template for PCR amplification of *ScFAR*, *ScWS*, and *ScFAE1* cDNAs from jojoba. PCR reactions were conducted with Phusion polymerase (New England Biolabs) using the 5′ and 3′ oligonucleotide pairs: (1) *FAR* 5′-TATATAGCGGCCGCAAAATGGAGGAAATGGGAAGC-3′ and 5′-TATATAGCGGCCGCTTTAGTTAAGAACGTGCTCTACGACACC-3′, (2) *WS* 5′-ATAGAATTCAAAATGGAGGTGGAGAAGGAGCTAAAGACC-3′ and 5′-AATAAGTCGACTCACCACCCCAACAAACCCAATTTC-3′, and (3) *FAE1* 5′-TATATAGCGGCCGCAAAATGAAGGCCAAAACAATCACAAAC-3′ and 5′-TATATAGCGGCCGCTTCTACGAAGCGATAGGTGCGATTTTAG-3′ (added restriction enzyme sites are underlined). The *ScWS* cDNA was ligated under control of the seed-specific soybean glycinin-1 promoter in the *Eco*RI/*Xho*I sites of the binary vector pBinGlyRed2^12^ as an *Eco*RI/*Sal*I-digested fragment to generate pBinGlyRed2-ScWS. The *ScFAR* and *ScFAE1* PCR products were ligated as *Not*I restriction enzyme fragments downstream of the seed-specific soybean glycinin-1 promoter and upstream of the glycinin-1 3′UTR into the corresponding site of the vector pKMS3^12^, resulting in *FAR* or *FAE1* expression cassette. The *FAR* cassette was cloned as an *Asc*I fragment into the *Mlu*I site of pBinGlyRed2-ScWS to generate pFWS2. An *nptII* kanamycin resistance gene under control of the *nos* promoter was amplified from pART27^30^ and ligated as an *Asc*I fragment into the corresponding site of pFWS2 to generate pFWS2-Kan. Alternatively, the *ScFAE1* cassette was digested from pKMS3 as an *Asc*I fragment and ligated into the corresponding site of pBinGlyRed2-ScWS to generate pFWS3. The *nptII* marker above was subsequently cloned as a *Kpn*I fragment into the corresponding site of the DsRed marker of pFWS3 to generate pFWS3-Kan. The resulting pFWS2-Kan and pFWS3 vectors contain the *DsRed* gene under control of the cassava mosaic virus promoter for selection of transgenic seeds, and the pFWS2-Kan and pFWS3-Kan vectors contain *nptII* for kanamycin selection of transgenic plants.

#### Preparation of pFWS3-LaFAE1

The *Lunaria annua FAE1*^27^ was amplified from plasmid pBluescript II SK+/*LaFAE1* using the oligonucleotides (added restriction sites are underlined): 5′-TATATTGCGGCCGCAAAATGACGTCTGTGAACGTAAAACTCC-3′ and 5′-TATATTGCGGCCGCTTAGGACCGACCGTTTTGGACAG-3′. The product was digested with NotI and cloned downstream of the glycinin-1 promoter and upstream of the glycinin-1 3′UTR. The resulting vector contained AscI restriction sites that flanked the glycinin-1 promoter and 3′UTR. Using this restriction site the entire cassette containing the promoter, gene and 3′UTR was used to replace the *ScFAE1* cassette in pFWS3 to generate pFWS3-LaFAE1.

#### Preparation of pBinGlyBar1-CsFAD2 HP

The seed-specific camelina *FAD2* RNAi suppression construct pBinGlyBar1-cFAD2 HP was prepared as described previously^12^.

#### Preparation of pFWS2-Hyg and pFWS3-Hyg

The DsRed selectable marker was replaced with a hygromycin resistance cassette by first digesting plasmid pMDC32^31^ with *Ase*I, which released the hygromycin cassette, then fragments were incubated with T4 DNA polymerase and dNTPs. The blunt-ended hygomycin cassette was isolated by DNA gel electrophoresis and purified using the Geneclean kit (MP Biomedicals, Solon, OH). The three-gene wax vector pFWS3 was digested with *Afl*II to release the *DsRed* marker, then fragments were blunted with T4 DNA polymerase and the vector fragment isolated and purified as described above. The hygromycin resistance cassette was ligated into the purified vector to produce pFWS3-Hyg, and orientation and copy number of the hygromycin cassette was confirmed using a combination of restriction mapping and DNA sequencing. The *ScFAE1* gene was then removed by digesting plasmid pFWS3-Hyg with *Asc*I, gel purifying the vector fragment, and self-ligation. The final plasmid (pFWS2-Hyg) was subjected to DNA sequencing to verify removal of the *ScFAE1* gene.

### *Agrobacterium* strains and plant transformation

The super-virulent *Agrobacterium* strain AGL1^32^ was used for crambe transformation, while strain GV3101(pMP90) was used for camelina and carinata transformations. Crambe transformation was performed using either hypocotyls explants according to the method described^15^ or the cotyledonary node method as developed for sugar beet transformation^33^ with minor modifications, essentially as described^34^. When using cotyledonary nodes as explants for crambe transformation, seeds were surface-sterilized with 70% (v/v) ethanol for 30 sec, followed by 3% (w/v) sodium hypochlorite with shaking at 42.5  °C for 20 min, and rinsed thoroughly with sterile water. The sterilized seeds were then placed onto the germination medium consisting of MS salts^35^ and vitamins, 2% (w/v) sucrose and 0.8% (w/v) Phytoblend (Caisson Labs) at pH 5.8 in plastic containers (50 seeds/container) and incubated at 7  °C overnight in the dark, followed by a dark period at 24 ^o^C for 3 days, and a further culture at 24  °C in 16 h photoperiod with a light intensity of 33 μmol·m^2^·s^−1^ for another 3 days prior to transformation. Camelina transformation was performed according to the floral *Agrobacterium* vacuum infiltration method described^12^,^16^. Carinata transformation was carried out using cotyledonary petiole explants, essentially as described^12^ with selection using 5 mg/L hygromycin.

### Screening of stable transgenic lines for seed wax ester content and composition

Total lipids were extracted from wild type and engineered seeds from the three oil crops using variations on the method described by Bligh and Dyer^36^ (crambe, camelina) or using hexane (carinata) followed by solid phase extraction or thin layer chromatographic (TLC) separation of wax ester (WE) and triacylglycerol (TAG) fractions. The total amount of each fraction and the fatty acid and fatty alcohol compositions of each were determined by gas chromatography after transesterification with added internal standards for quantification. Detailed analytical methods for seeds for each crop are provided below.

Crambe: For screening stable transgenic lines, the half-seed technique^28^ was used in all generations. Seeds were removed from siliques and surface-sterilized using 15% calcium hypochlorite for 15–20 min and rinsed thoroughly with sterile water. The surface-sterilized seeds were then placed on the germination medium in a climate-controlled chamber as described^15^. After culture for ~15 h, seed coats were removed by dissection under microscope, and the larger outer cotyledon was excised for wax ester analysis, while the rest of the seeds were continuously maintained on the culture medium until planted in pots. Seedlings with the highest WE content were planted in the greenhouse or biotron depending on season. Alternatively, seeds were soaked in water for 2 h. Partial cotyledons were then cut off with a fine knife under the microscope. After cutting, the chipped fragment of the cotyledon was immediately used for oil composition analysis by GC as described below. The remaining part of the seed was sown directly into a pot with well-irrigated soil in the greenhouse where a cover of gas-permeable mesh on the top of each pot was used to protect the emerging seedlings from insect infestation.

For total lipid extraction, excised cotyledons were homogenized in a glass tube with 1.9 ml of methanol:chloroform (2:1) and 0.6 ml of 0.15 M acetic acid. To the homogenate, 0.6 ml of chloroform and 0.6 ml of water were added, mixed and centrifuged for 3 min at 3,000 rpm^28^. The lower phase was transferred into a screw cap tube and dried under N_2_. The residue was resuspended in 60 μl of chloroform for TLC and GC analyses. Approximately 40 μl of lipid extract was analyzed on TLC silica gel plates (15 cm × 20 cm or 20 cm × 20 cm, Merck) in a solvent system of hexane:ethyl ether:acetic acid (90:10:1 v/v/v) to separate WE and TAG. The areas corresponding to the reference sample (standard jojoba wax ester, visualized by exposure to iodine) containing WE and TAG were scraped and collected in a screw-cap tube with addition of 200 μl methanol to remove the water from the gel under nitrogen stream. Two ml methylation solution (2% sulphuric acid in dry methanol) was immediately added and the samples were transesterified at 95 °C for 45 min. After methylation, 0.5 ml hexane, 2 ml water and internal standard 17:0 alcohol were added. After brief vortexing, the samples were centrifuged at 3000 rpm for 3 min. The hexane phase containing fatty alcohol and FAME of the wax part or FAME of the TAG part was transferred into a GC vial for analysis using a Shimadzu GC-17A GC fitted with a CP-Wax 58 column as described^28^. Peaks were identified according to their relative retention time in comparison with external standards run on the same instrument. The WE content was calculated based on the peak areas. Total lipids in crambe seeds were determined as described^37^.

Camelina: DsRed fluorescent seeds from T_1_ plants of ≥10 independent transgenic lines for each construct were screened by TLC to identify wax producing lines. Lines containing waxes were further analyzed by GC following direct transesterification of homogenized seeds to quantify fatty alcohol content (as described below). Three independent lines from transformants containing the highest seed WE content were selected for advancing to homozygosity and for more complete characterization of WE content and composition and agronomic properties. Two homozygous lines from the pFWS3-LaFAE1 transformants with the highest seed WE content were retransformed with the pBinGlyBar1-cFAD2 HP construct, and transgenic lines were selected by glufosinate resistance as described^12^. Seeds from T_1_ plants from >15 independent transformants were screened by GC following methylation of the seed oil to measure fatty acid and fatty alcohol content of seeds as described below. For initial screening of transformants, 3–4 seeds were homogenized with a glass rod in 200 μl of heptane:ethyl ether (50:50 v/v). Approximately 25 μl of the extract was analyzed on TLC silica plates in a solvent system of heptane:ethyl ether:acetic acid (60:40:1 v/v/v) with TAG and WE standards. Lipids were visualized by staining in iodine vapor. In addition, ~5 homogenized seeds were methylated in 2.5% (v/v) sulphuric acid in methanol as described^12^ and analyzed using an Agilent 7890 GC fitted with an Agilent HP-INNOWax column (30 m length × 0.25 mm inner diameter, 0.25 μm film thickness), H_2_ carrier gas, and flame ionization detection. The oven temperature was programmed from 185 °C (1 min hold) to 235 °C at 7 °C/min (5 min hold) and to 240 °C at 10 °C/min (5 min hold). Fatty acid methyl ester and fatty alcohol were identified by mobility relative to authentic standards and their structures confirmed by GC-MS using an Agilent 7890 gas chromatograph interfaced with an Agilent 5973 mass selective detector. For quantification of WE and TAG in seeds of homozygous lines, seeds (~25 mg) were weighed in glass screw cap tubes (13 × 100 mm), and 175 μl of triheptadecanoin (10 mg/ml in toluene) and 70 μl of myristitic acid-myristoyl alcohol WE (5 mg/ml in toluene), both purchased from NuChek Prep, were added as internal standards. Total lipids were then extracted from seeds in 3 ml of chloroform:methanol (1:2 v/v) and grinding with an Omni THQ tissue homogenizer. After 1 h incubation with shaking at 25 °C, 1 ml of chloroform and 1.8 ml of water were added to the extract. Following thorough shaking and centrifugation, the organic layer was recovered. The organic layer was dried under N_2_ and re-suspended in 1 ml of heptane. This was applied to an activated silica solid phase extraction column (Supelco Supelclean LC-Si, 3 ml column; Sigma-Aldrich, Saint Louis, MO USA) equilibrated with heptane. One ml of heptane was subsequently applied to the column. The WE fraction was then eluted with 2.5 ml of heptane:ethyl ether (95:5 v/v), and followed by 2.5 ml of heptane:ethyl ether (80:20 v/v) to eluted TAGs. An aliquot of each fraction was analyzed by TLC (as described above) to confirm separation of these fractions. The WE and TAG eluents were then transesterified and analyzed by GC as described above and quantified relative to the added internal standards.

Carinata: The initial screening of transgenic T_1_ lines for presence of WE was carried out using single seed TLC by lightly crushing individual seeds placed in the wells of a 96 well microtiter plate. Two drops of hexane were added to each well and extracted lipids were transferred to a silica gel TLC plate using a multichannel pipette set to a 3 μl volume. Plates were developed in hexane:ethyl ether:acetic acid (140:30:3 v/v/v), and lipids were visualized by exposure to iodine vapors. Seed lipids were extracted by grinding 10 pooled seeds in hexane with a 10 min incubation at 42 °C. Sediment was removed by centrifugation and the clear hexane phase was evaporated to dryness under N_2_. Lipids were dissolved in hexane and stored at −20 ^o^C until used for analysis. WE and TAG were first separated by TLC on the silica gel plates using the same solvent system as described above for single seed analysis. Areas containing lipids, visualized using iodine vapors, were scraped from the plates and directly methylated along with internal standards to generate fatty acid methyl esters and fatty alcohols using 1 M hydrochloric acid in methanol, with heating at 80 °C for 2 h. Extracted fatty acid methyl esters and fatty alcohols were analyzed simultaneously by GC on a DB23 column (30 m length × 0.25 mm inner diameter; Agilent) as described^25^. Identity of the fatty alcohols was verified by GC-MS of their trimethylsilyl-ether derivatives.

### Wax ester profiling by nanoESI-MS/MS

Quantitative wax ester profiling of oil from total lipid extracts of engineered crambe, camelina, and carinata seeds was performed following a previously published direct infusion nanoESI-MS/MS method^38^ with minor modifications. WE molecular species (484 total) carrying acyl chain combinations from 16:0 to 26:1 were monitored. Detector signals below 50 counts per second were defined as background noise. The WE profiles represent amounts of the 20 (Fig. 5d) or 10 (Fig. 3b) WE most abundant molecular species [mol %] relative to the sum of these molecular species.

### Crossing of crambe wax ester lines with *FAD2* down-regulated lines

Two previously described transgenic crambe lines with *CaFAD2* knocked down by RNAi (*CaFAD2*-RNAi) driven by the napin promoter^28^ were crossed with two transgenic lines transformed co-expressing the *ScFAR* and *ScWS* genes. For the crossing, only unopened floral buds were selected, while all open flowers and non-selected buds were removed from the inflorescences on the mother plants. The selected flower buds were manually opened and anthers were removed. Styles and stigmas were exposed and used in pollinations. After 24 h, from the father plants, open flowers having matured anthers with visible pollen shedding were chosen. Anthers were tapped on the stigmas of the mother plants to cover them with pollen grains. Subsequently, the artificially pollinated flowers on mother plants were marked. Each of the plants was used both as father and mother in reciprocal crosses. The F_1_ seeds were sown directly in the soil for bringing to F_2_ for oil analysis. For the F_2_ seeds, the wax ester analysis was carried out using the half-seed technique as described above.

### Crambe and camelina seed germination test

For crambe, 150 seeds without siliques were put onto the wet filter paper in Petri dishes which were placed in the dark in the climate-controlled chamber at a temperature of 25 °C. The germination results were recorded at 3, 5, and 7 days after sowing and the test was repeated three times. For camelina, ninety seeds were sown directly in the soil with three replicates. The germination results were recorded after one week.

### Crambe field trial

A field trial on crambe was conducted in southern Sweden. The planting area was 75.6 m^2^ for the transgenic crambe with the *FAR* and *WS* genes, 25.2 m^2^ for the transgenic crambe with the *FAR*, *FAE* and *WS* genes, and 65.7 m^2^ for the wild type. The crambe plants were grown under insect net to prevent bees from spreading GM-pollens as required by the EU court. The wax esters were extracted from the seeds and analyzed as described above.

## Additional Information

**How to cite this article**: Zhu, L. *et al.* Dedicated Industrial Oilseed Crops as Metabolic Engineering Platforms for Sustainable Industrial Feedstock Production. *Sci. Rep.* **6**, 22181; doi: 10.1038/srep22181 (2016).

## Supplementary Material

Supplementary Dataset 1

## Figures and Tables

**Table 1:** Agronomic traits of wax ester transgenic lines from field trial (T_5_ generation) and greenhouse (T_6_ generation) evaluations of crambe.

Line	1000 seed weight (g)	Seed yield	Yield in relative to wild type (%)*	Wax ester content (mg/g seed)**	Oil content (%) ***	Oil content relative to wild type (%)*	Germination (%)
field trial		kg/ha					
Wild type	6.0 ± 0.1c^1^	1850	100	n.d.	44.3 ± 5.0a	100	100
2 gene Line	6.9 ± 0.2b	1366	74	79.6 ± 2.3a	41.9 ± 0.6a	95	90
3 gene line	8.8 ± 0.2a	350	19	54.5 ± 3.7b	30.3 ± 0.6b	68	85
greenhouse		g/plant					
Wild type	7.0 ± 0.2b	20.9 ± 4.2a	100	n.d.	40.3 ± 4.5a	100	100
2 gene line 1	7.2 ± 0.2b	19.1 ± 2.4a	91	89.0 ± 11.0a	38.7 ± 4.1a	96	100
2 gene line 2	7.3 ± 0.2b	18.4 ± 5.2ab	88	80.9 ± 12.3a	38.0 ± 3.3a	94	100
3 gene line	9.5 ± 1.0a	8.8 ± 4.9b	42	54.7 ± 14.3a	32.2 ± 5.0a	80	87

Note: 2 gene lines: *ScFAR/ScWS;* 3 gene line: *ScFAR/ScWS/ScFAE1*. *Results are normalized to 100% for wild type. **Three-seed pooled sample and three biological replicates. ***20-seed pooled sample, three biological replicates for the field trial material and 4 biological replicates for the greenhouse material. ^1^Different letters followed by the figures in the columns of 1000 seed weight, wax ester content and oil content both for field and greenhouse data and seed yield for greenhouse data (n = 4 plants) indicate significant differences at p = 0.05, analyzed using the Kyplot program with the Tukey test. n.d., not detected.
