# Supplementary material for: Dedicated Industrial Oilseed Crops as Metabolic Engineering Platforms for Sustainable Industrial Feedstock Production
Source: Sci Rep. 2016 Feb 26;6:22181. doi: 10.1038/srep22181 (PMC4768164; doi:10.1038/srep22181)
Supplement: Supplementary Dataset 1 [file srep22181-s1.doc]

**Dedicated Industrial Oilseed Crops as Metabolic Engineering Platforms for Sustainable Industrial Feedstock Production**

Li-Hua Zhu^1*^, Frans Krens^2^, Mark A. Smith^3^, Xueyuan Li^1^, Weicong Qi^2^, Eibertus N. van Loo^2^, Tim Iven^4^, Ivo Feussner^4,5,6^, Tara J. Nazarenus^7^, Dongxin Huai^7,8^, David C. Taylor^3^, Xue-Rong Zhou^9,10^, Allan G. Green^10^, Jay Shockey^11^, K. Thomas Klasson^11^, Robert T. Mullen^12^, Bangquan Huang**^1^**^3^, John M. Dyer^14^ and Edgar B. Cahoon^7*^

\

**Supplementary Table S1.** Wax ester content in seed lipid from different generations of transgenic crambe lines with different constructs

| Line | Seed number | Minimum (%) | Maximum (%) | Mean |
| --- | --- | --- | --- | --- |
| 2 gene construct |  |  |  |  |
| T_1_ | 17 | 1.5 | 31.0 | 11.3 |
| T_2_ | 32 | 2.5 | 50.7 | 21.8 |
| T_3_ | 150 | 3.8 | 54.4 | 29.5 |
| T_4_ | 48 | 11.5 | 48.5 | 29.0 |
|  |  |  |  |  |
| 3 gene construct |  |  |  |  |
| T_1_ | 20 | 0.4 | 50.7 | 23.7 |
| T_2_ | 58 | 6.6 | 55.0 | 32.8 |
| T_3_ | 122 | 13.9 | 52.1 | 29.5 |
| T_4_ | 60 | 14.4 | 51.6 | 30.5 |
|  |  |  |  |  |
| Wild type | 20 | 0 | 0.6 | 0.3 |

**Supplementary Table S2.** Wax ester content in the seed oil of hybrids (F_2_) between the wax ester line and *CaFAD2*-RNAi line of crambe grown in greenhouse

| Seed number | WE content (%) | | |
| --- | --- | --- | --- |
|  | Line 1 | Line 2 | Line 3 |
| 1 | 41.5 | 0.6 | 28.9 |
| 2 | 29.9 | 31.2 | 28.8 |
| 3 | 35.0 | 30.6 | 29.5 |
| 4 | 32.7 | 0.5 | 23.7 |
| 5 | 33.2 | 17.8 | 26.8 |
| 6 | 23.6 | 36.2 | 24.2 |
| 7 | 33.3 | 34.1 | 23.1 |
| 8 | 35.9 | 37.7 | 26.3 |
| 9 | 28.7 | 37.1 | 22.6 |
| 10 | 33.7 | 27.4 | 24.5 |
| 11 | 18.0 | 29.0 | 22.4 |
| 12 | 31.2 | 22.3 | 20.0 |
| 13 | 24.7 | 18.1 | 20.5 |
| 14 | 31.5 | 6.5 | 15.6 |
| 15 | 26.5 | 45.7 | 19.4 |
| 16 | 32.4 | 0.5 | 21.3 |
| 17 | 26.5 | 21.0 | 25.3 |
| 18 | 32.6 | 6.7 | 24.0 |
| 19 | 36.8 | 35.9 | 23.3 |
| 20 | 31.6 | 25.0 | 32.5 |
| 21 | 27.6 | 38.5 | 25.5 |
| 22 | 21.1 | 28.6 | 19.2 |
| 23 | 33.7 | 12.7 | 21.7 |
| 24 | 30.1 | 7.2 | 21.1 |
| Mean | 30.5 | 23.0 | 23.8 |


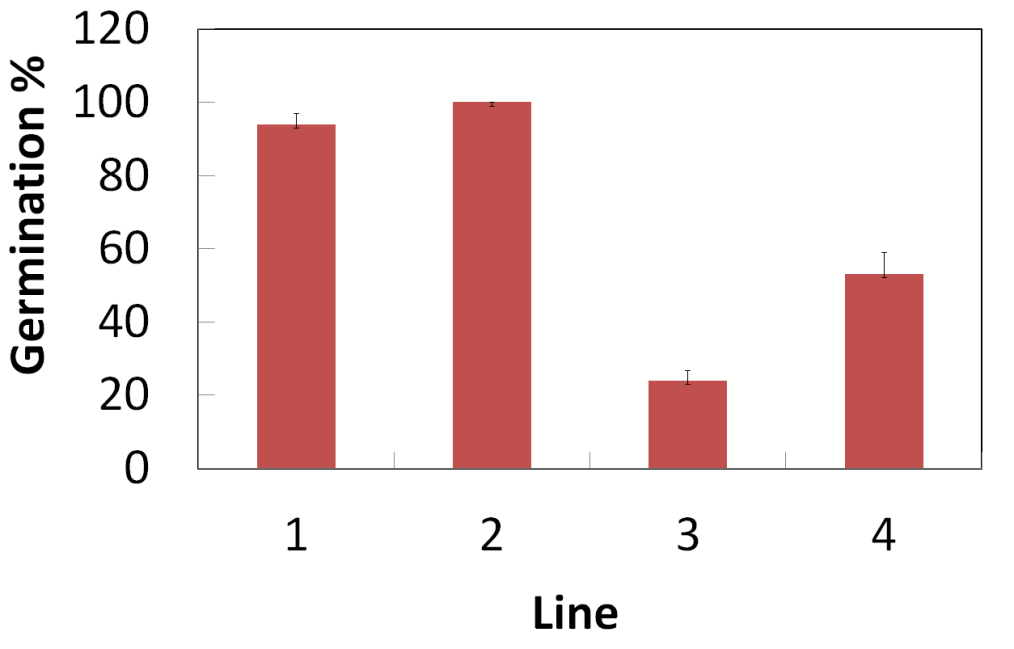


**Supplementary Figure S1**. Germination results of seeds of different camelina lines. 1= wild type. 2=*CsFAD2*-RNAi line. 3=*ScFAR*/*ScWS*/*LaFAE1* line. 4= *ScFAR*/*ScWS*/*LaFAE1*/*CsFAD2*-RNAi line. (n=3 biological replicates ± SD)
